# Supplementary material for: Placental growth factor modulates endothelial NO production and exacerbates experimental hepatopulmonary syndrome
Source: JHEP Rep. 2024 Dec 10;7(3):101297. doi: 10.1016/j.jhepr.2024.101297 (PMC11840504; doi:10.1016/j.jhepr.2024.101297)
Supplement: Multimedia component 1 [file mmc1.pdf]

Placental growth factor modulates endothelial NO production and exacerbates experimental hepatopulmonary syndrome

Fabien Robert, Feriel Benchenouf, My Ngoc Ha, Alessandra Cuomo, Mina Ottaviani, Maxime Surbier, Raphaël Thuillet, Corinne Normand, Florent Dumont, Céline Verstuyft, Frederic Fiore, Frederic Guinut, Marc Humbert, Audrey Coilly, Emmanuel Gonzales, Olivier Sitbon, Ly Tu, Christophe Guignabert, Laurent Savale

Table of contents

Fig. S1……………………………………………………………………………………2

Fig. S2……………………………………………………………………………………3

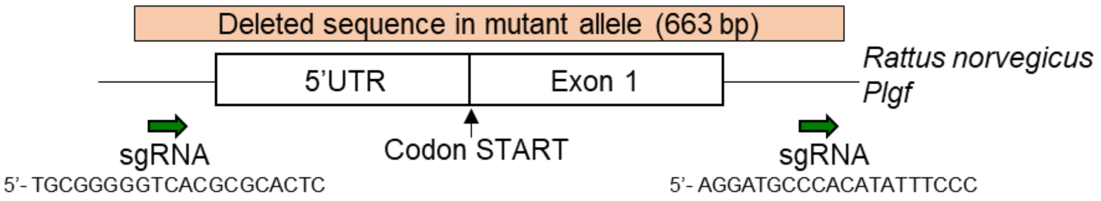

Fig. S1. Generation of the *Plgf*-deficient rat model.

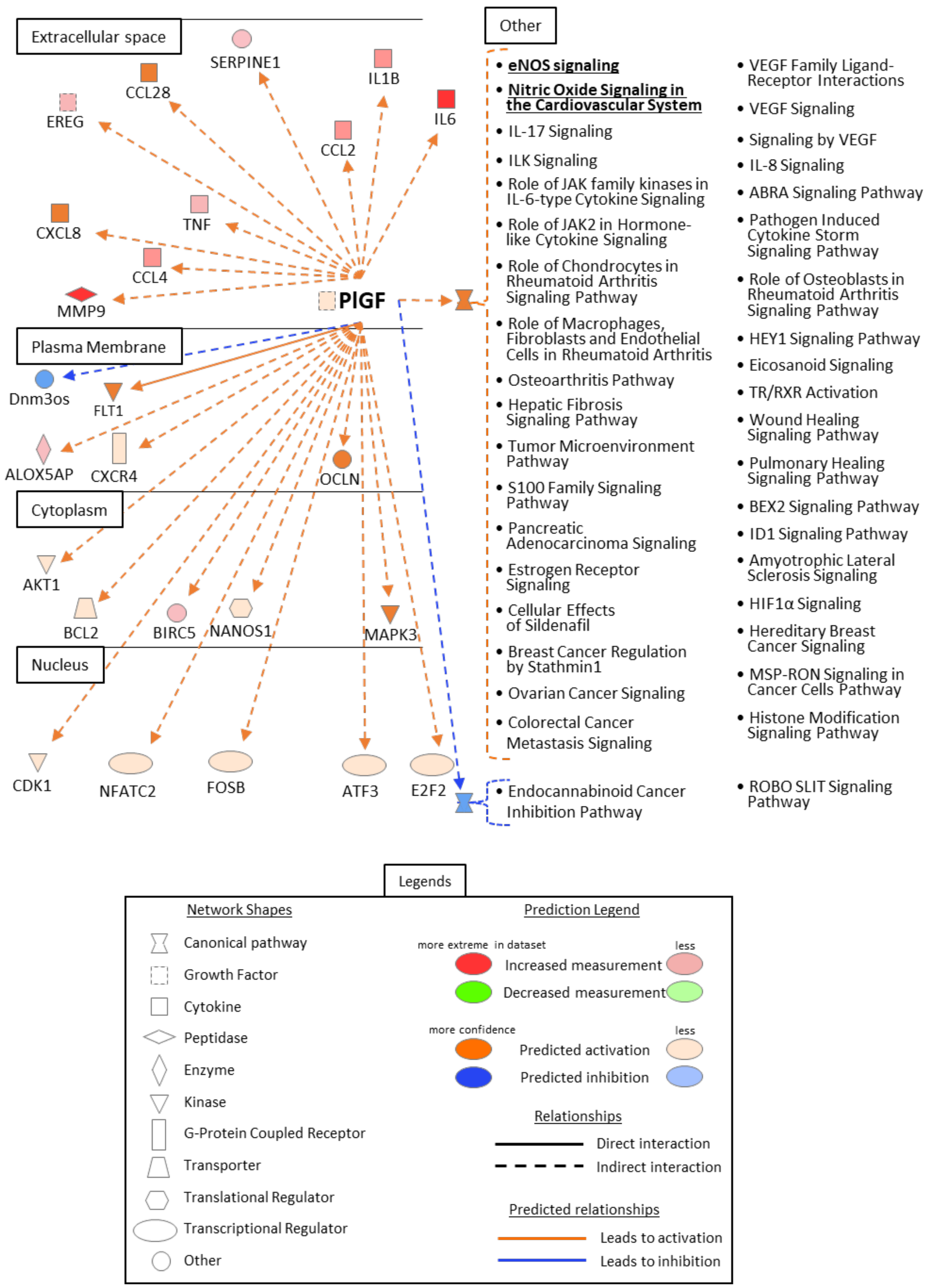

Fig. S2. Interaction between PlGF and the pulmonary transcriptome of CBDL rats using My Pathway analysis (IPA tool, QIAGEN). Cut-off: p-value<0.05, fold-change>|1.5|. The predicted relationships are represented with either full-lined arrows for direct or dotted arrows for indirect interaction. The predicted relationships that lead to activation or inhibition of the downstream targets or gene sets are shown in orange and blue, respectively. Upregulated genes are represented in red and downregulated genes in green. Predicted activated or inhibited genes and gene sets are shown in orange and blue, respectively.
