# Supplementary material for: Placental growth factor modulates endothelial NO production and exacerbates experimental hepatopulmonary syndrome
Source: JHEP Rep. 2024 Dec 10;7(3):101297. doi: 10.1016/j.jhepr.2024.101297 (PMC11840504; doi:10.1016/j.jhepr.2024.101297)
Supplement: Multimedia component 2 [file mmc2.docx]

**JHEP Reports**

**CTAT methods**

Tables for a “Complete, Transparent, Accurate and Timely account” (CTAT) are now mandatory for all revised submissions. The aim is to enhance the reproducibility of methods.

- Only include the parts relevant to your study
- Refer to the CTAT in the main text as ‘Supplementary CTAT Table’
- Do not add subheadings
- Add as many rows as needed to include all information
- Only include one item per row
  1. **Antibodies**

| **Name** | **Supplier** | **Cat no.** | **Clone no.** |
| --- | --- | --- | --- |
| NOS3 | BD Biosciences | 610297 | 3/eNOS/NOS Type III |
| p-NOS3 | Santa Cruz Biotechnology | Sc-81510 | 15E2 |
| β-actin | Sigma-Aldrich | A3854 | AC-15 |

- 1. **Cell lines**

| **Name** | **Supplier** | **Passage no.** |
| --- | --- | --- |
| Primary human pulmonary microvascular endothelial cells | Our laboratory | <5 |

- 1. **Organisms**

| **Name** | **Supplier** | **Strain** | **Sex** | **Age** | **Overall n number** |
| --- | --- | --- | --- | --- | --- |
| Rats | Janvier Labs | Sprague- Dawley | Male | 8 weeks old | 58 |

- 1. **Sequence based reagents**

| **Name** | **Sequence ID** | **Supplier** |
| --- | --- | --- |
| PlGF siRNA | HSS143278 | Thermo Fisher Scientific |
| PlGF mRNA RT-qPCR | Rn00677739_m1 | Thermo Fisher Scientific |

- 1. **Biological samples**

| **Description** | **Source** |
| --- | --- |
| Patient serum | Hôpital Bicêtre  Hôpital Paul Brousse  Centre de Ressource Biologique Paris Saclay  Etablissement Français du Sang |

- 1. **Deposited data**

| **Name of repository** | **Identifier** |
| --- | --- |
| GEO NCBI database | GSE278353 |

- 1. **Software**

| **Software name** | **Manufacturer** | **Version** |
| --- | --- | --- |
| SPSS Statistics | IBM, SPSS Inc. | Version 29 |
| GraphPad Prism | GraphPad Software | Version 10.0.0 |
| R | The R Foundation for Statistical Computing | R version 4.3.2 (2023-10-31) |
| Rstudio software | Posit, PBC | 2024.4.2.764 |
| IPA | Qiagen IPA | Fall Release Q3 2024 |

- 1. **Other (*e.g*. drugs, proteins, vectors etc.)**

| **Name** | **Supplier** | **Cat no.** |
| --- | --- | --- |
| rHu PlGF | Bio-Techne | 264-PGB |

- 1. **Please provide the details of the corresponding methods author for the manuscript:**

| **Dr Ly TU ly.tu@inserm.fr** |
| --- |
