# Supplementary material for: Placental growth factor modulates endothelial NO production and exacerbates experimental hepatopulmonary syndrome
Source: JHEP Rep. 2024 Dec 10;7(3):101297. doi: 10.1016/j.jhepr.2024.101297 (PMC11840504; doi:10.1016/j.jhepr.2024.101297)
Supplement: Multimedia component 4 [file mmc4.pdf]

JHEP|Reports

Placental growth factor modulates endothelial NO production and exacerbates experimental hepatopulmonary syndrome

Fabien Robert[1,2], Feriel Benchenouf[1,2], My Ngoc Ha[1,2], Alessandra Cuomo[1,3], Mina Ottaviani[1,2], Maxime Surbier[1,2], Raphaël Thuillet[1,2], Corinne Normand[1,2], Florent Dumont[1,2], Céline Verstuyft[4], Frederic Fiore[5], Frederic Guinut[6], Marc Humbert[1,2,7], Audrey Coilly[8,9], Emmanuel Gonzales[9,10], Olivier Sitbon[1,2,7], Ly Tu[1,2], Christophe Guignabert[1,2,†], Laurent Savale[1,2,7,*,†]

JHEP Reports **2025**. vol. 7 | 1–10

Check for updates

Background & Aims: Hepatopulmonary syndrome (HPS) results from portal hypertension, with or without cirrhosis, and is marked by pulmonary vascular dilations leading to severe hypoxemia. Although placental growth factor (PlGF) is important for vascular growth and endothelial function, its role in HPS is unclear. This study investigated the involvement of PlGF in experimental models of HPS and in patients.

Methods: Circulating PlGF levels were measured in 64 controls and 137 patients with liver disease, with or without HPS. Two rat models, common bile duct ligation (CBDL) and long-term partial portal vein ligation (PPVL), were used. *Plgf*-knockout (*Plgf*$^{-/-}$) rats were generated using CRISPR-Cas9. Lung RNA-sequencing analysis was performed in the CBDL model. The effects of PlGF on endothelial nitric oxide synthase (eNOS) activity in human pulmonary microvascular endothelial cells were also investigated.

Results: Circulating PlGF levels were significantly higher in patients with cirrhosis compared with healthy controls (29.4 ± 1.2 *vs.* 20.2 ± 0.8 pg/ml, p <0.0001), but no difference were found between patients with and without HPS. PlGF levels were not elevated in patients with extrahepatic portal hypertension. In *Plgf*$^{-/-}$ rats, there was a protective effect against CBDL-induced HPS, whereas PPVL-induced HPS severity remained unchanged. RNA sequencing coupled with ingenuity pathway analysis identified significant interactions between PlGF and pulmonary eNOS activity. Following CBDL, *Plgf*$^{-/-}$ rats showed decreased pulmonary eNOS activity and reduced circulating nitric oxide metabolites. *In vitro*, PlGF stimulation enhanced eNOS activity in human pulmonary microvascular endothelial cells, whereas PlGF knockdown led to a decrease.

Conclusions: These findings indicate that PlGF aggravates cirrhosis-induced HPS through modulation of pulmonary eNOS activity, and is not involved in HPS from extrahepatic portal hypertension.

© 2024 The Author(s). Published by Elsevier B.V. on behalf of European Association for the Study of the Liver (EASL). This is an open access article under the CC BY license (http://creativecommons.org/licenses/by/4.0/).

Introduction

Hepatopulmonary syndrome (HPS) poses a significant challenge in chronic liver diseases, marked by liver dysfunction, intrapulmonary vascular dilatations (IPVDs) and arterial hypoxemia.[1] HPS notably impacts the quality of life and survival of patients, making it of crucial clinical concern.[2–5] Although commonly associated with cirrhosis, HPS can also occur independently, particularly in cases of extrahepatic portal hypertension or congenital portosystemic shunts.[1,6,7] This heterogeneity highlights the complex interplay between the portal and pulmonary circulatory systems, suggesting multifaceted pathophysiological mechanisms driving HPS.

Unfortunately, the cellular and molecular mechanisms underlying the onset and progression of HPS remain poorly understood, contributing to the lack of curative treatments. As a result, liver transplantation remains the only effective therapeutic option.[8] Placental growth factor (PlGF) has a role in regulating vascular tone,[9–14] angiogenesis,[15–19] and inflammatory processes[20–22] in various pathological contexts. This member of the vascular endothelial growth factor (VEGF) family is abundantly expressed in conditions affecting the liver and lungs.[22–25] Blocking PlGF with antibodies has been shown to reduce IPVDs and hypoxemia in cirrhotic mice with HPS by modulating pulmonary inflammation and angiogenesis.[22]

However, despite this promising evidence, it remains unclear whether PlGF can serve as a true therapeutic target in HPS. Most studies have focused on the common bile duct ligation (CBDL) model and patients with cirrhosis. There is a lack of data on the involvement of PlGF in patients with portal hypertension without cirrhosis, or in the partial portal vein ligation (PPVL) model of HPS associated with extrahepatic portal hypertension. Given the ubiquitous nature of portal hypertension in chronic liver diseases, irrespective of cirrhosis, understanding its role in the pathogenesis of HPS is crucial. Exploring the dynamics of PlGF across various hepatic

* Corresponding author. Address: INSERM UMR_S 999, Team 1, Faculté de Médecine, Université Paris-Saclay, Bâtiment de recherche (2e étage), 63 rue Gabriel Péri, 94276 Le Kremlin Bicêtre, France. Tel: +33 1 40 94 88 33.
E-mail address: laurent.savale@aphp.fr (L. Savale).
† These authors contributed equally to the manuscript.
https://doi.org/10.1016/j.jhepr.2024.101297

contexts is also essential for developing effective targeted therapeutic strategies.

To bridge this gap, our study investigated PlGF expression in patients across different contexts of portal hypertension, both with or without cirrhosis, and its association with HPS. In addition, we examined the role of PlGF in the development of HPS using two complementary experimental models: the cirrhosis-related CBDL model[26] and the long-term PPVL model, which induces portal hypertension without cirrhosis.[27] By analyzing PlGF levels across these distinct hepatic conditions, we investigated its specific contributions to the pathophysiology of HPS. To gain further insight into the role of PlGF in pulmonary vascular dysfunction, we conducted *in vitro* studies using primary cultures of human pulmonary microvascular endothelial cells (PMECs).

Material and methods

Cohort data collection

This study was approved by the CPP Ile-de-France – VII ethics committee (approval number: CO 10-003) and conducted in accordance with the Declaration of Helsinki. All patients gave informed consent for data and sample collection. In total, 130 patients diagnosed with cirrhosis (with or without HPS) between 2017 and 2024 were included in this study. In addition, samples from seven patients with portal cavernoma without cirrhosis were also included. Patients with combined portopulmonary hypertension were excluded. Lastly, 64 control samples from healthy individuals were obtained through the Etablissement Français du Sang (EFS).

Cirrhosis was diagnosed based on clinical and laboratory findings or liver biopsy, with severity assessed using the Child-Pugh or Model for End-Stage Liver Disease (MELD) classifications. Portal hypertension was diagnosed using hemodynamic measurements or suggestive signs, such as esophageal varices, splenomegaly, thrombocytopenia, presence of portosystemic collaterals, ascites, and encephalopathy.

The diagnosis of HPS in patients with cirrhosis was confirmed by the presence of IPVDs, assessed using contrast-enhanced echocardiography, along with abnormal arterial oxygenation defined by an alveolar–arterial oxygen gradient (A-aO_2) >15 mmHg (or >20 mmHg in patients >64-years old).[28]

Serum samples from healthy controls, patients with cirrhosis, or patients with portal cavernoma were stored at –80 °C. Circulating PlGF levels were quantified using the SimplePlex Ella™ microfluidic platform (Protein Simple) following the manufacturer's instructions.

Generation of *Plgf*-deficient rats

Gene knockout of *Plgf* (from the Ensembl browser [www.ensembl.org]: no. ENSRNOG00000005650) was achieved by deleting a 663-base pair (bp) sequence. The suppressed region corresponded to the 5′-untranslated region (UTR), start codon, and Exon 1 of *Plgf* (Pgf-201 ENSRNOT00000007790.5 transcript) (Fig. S1). Two CRISPR guides were selected to induce the deletion: a single guide (sg)RNA (5′-TGCGGGGGTCACGCGCACTC) targeting the region located 56 bp before the 5′UTR and a sgRNA (5′- AGGATGCCCACATATTTCCC) targeting the region located 119 bp after Exon 1 of *Plgf*. Using CRISPR-Cas9 gene-editing technology, CRISPR guides and Cas9 mRNA were microinjected into RjHan:SD zygotes (RjHan:SD wild-type (WT) zygotes provided by Janvier Labs, Le Genest-Saint-Isle, France). The micro-injection mix comprised 15 ng/µl of Cas9 (mRNA) and 5 ng/µl of each sgRNA. The F0 founders (mosaics) were screened for the desired mutation using PCR and sequencing. These founders were then mated to RjHan:SD breeders to segregate the targeted allele. The N1 progeny were identified using PCR and sequencing. The international designation for this edited rat lineage is RjHan:SD-Pgfem1Ciphe/Rj.

In vivo experimental design

All animal care and procedures were approved by the French Animal Experimentation Ethics Committees (permit number: Apafis #23045). Male *Plgf*-deficient rats or their WT littermates (8-weeks old; Janvier Labs) underwent CBDL and PPVL surgery as previously described.[26,27,29,30] Analyses were performed 4 weeks post CBDL and 10 weeks post PPVL, because we previously demonstrated that HPS develops after these time points.[27] Age-matched Sham-operated control animals were also included for comparison.

Arterial oxygenation and intrapulmonary vascular dilations measurements in rats

Arterial blood gas analysis

Rats were anesthetized with 2% inhaled isoflurane in ambient air, and their body temperature was maintained at 37 °C. A tracheotomy was performed, and ventilation was adjusted using a Small Animal Ventilator (Harvard Apparatus, Holliston, MA, USA) to maintain the partial pressure of arterial carbon dioxide ($PaCO_2$) between 35 and 45 mmHg. Within 5 min of anesthesia induction, arterial blood samples were collected from the left carotid artery, and the partial pressure of arterial oxygen (PaO_2) was measured using the epoc® blood analysis system (Siemens Healthineers, Courbevoie, France). The A-aPO_2 was then calculated.

Contrast-enhanced echocardiography

A microbubble-based contrast agent was injected into each rat through a jugular vein catheter. The microbubbles (ranging from 10 µm to 40 µm) were larger than the average diameter of the pulmonary capillaries. The presence of microbubbles in the left ventricle after five cardiac cycles following injection indicated the presence of IPVDs. The signal was calculated by the difference in decibels (ΔdB) before and after injection in both the right and left ventricles. The signal ratio between the left and right ventricles was calculated to assess the severity of IPVDs. Detection was performed using the Vevo® 3100 LT echocardiography system (FUJIFILM VisualSonics Inc., Paris, France).

Systemic fluorescent microspheres dissemination

A total of 7×10^6 orange fluorescent microspheres (8 µm in diameter, Phosphorex) were injected into each rat through a jugular vein catheter. Under normal physiological conditions, the pulmonary capillaries trap the fluorescent microspheres, whereas the presence of IPVDs facilitates their dissemination into the systemic circulation. One minute after injection, the kidney was removed, and the number of fluorescent

microspheres in the kidney was quantified using flow cytometry with the Accuri^{TM} C6 instrument (BD Biosciences, Le Pont de Claix, France) as previously described.[27]

Liver function assessment

Portal vein venous flow was measured using hepatic Doppler ultrasound with the Vevo® 3100 LT echocardiography system (FUJIFILM VisualSonics Inc.) on anesthetized rats. Spleen and liver weights were recorded, and 5-μm liver sections were analyzed following Picro-Sirius Red staining. Blood samples were tested for common liver enzymes at the Service de Biologie Médicale (Marie-Lannelongue hospital, Le Plessis-Robinson, France).

Circulating PlGF levels, complete blood cell count, and nitrite/nitrate analysis in animal samples

Serum PlGF levels were quantified using an ELISA kit (E-CL-R0520, ElabScience, Houston, Texas, 77079, USA) following the manufacturer's instructions. Total white blood cell, neutrophil, lymphocyte, and monocytes counts were measured in blood samples using Element HT5 (HESKA distributed by Altorf, France). Nitrite and nitrate levels were quantified in serum samples using a colorimetric Nitrite/Nitrate Assay kit (23479, Sigma-Aldrich, Saint-Quentin-Fallavier Cedex, France) following the manufacturer's instructions.

RNA extraction and transcriptomic analysis

RNA extraction from lung biopsies was performed using the RNeasy® Mini Kit (QIAGEN, Courtaboeuf, France) following the manufacturer's protocol. A total of 250 ng of RNA was used for next-generation sequencing (NGS) library preparation using the Illumina Stranded mRNA Prep kit following the manufacturer's instructions. Data analysis was performed using R and RStudio software. Reads were independently mapped to the *Rattus norvegicus* genome mRatBN7.2 using the Rsubread package.[31] Genes were considered significantly upregulated with a fold-change >1.5, and downregulated with a fold-change <1.5, all with p <0.05. My pathway tool from Ingenuity pathway analysis (IPA) QIAGEN (QIAGEN Inc., https://digitalinsights.qiagen.com/IPA) was used to map all known direct and indirect interactions of PlGF with downstream targets or gene sets according to the IPA database. The previously generated dataset was overlaid onto this network. All raw and processed data have been submitted to the GEO NCBI database under accession number GSE278353.

In vitro experimental design

Human PMECs were isolated and cultured as previously described,[32] and used for early passages. Experiments were conducted in a serum-depleted medium (0.5% fetal calf serum). To analyze the effect of exogenous PlGF on human PMECs, synchronized cells were stimulated for 30 min or 24 h with the vehicle (PBS) or recombinant PlGF (Catalog 264-PGB, Bio-Techne, Minneapolis, MN, USA) at concentrations of 50 or 200 ng/ml. To suppress PlGF expression, synchronized cells were transfected using lipofectamine RNAiMAX with 100 nM of PlGF short interfering (si)RNA (HSS143278, Thermo Fisher Scientic, Villebon-sur-Yvette, France) or with a scrambled sequence, and the cells were studied within 1 day after transfection.

Western blot and real-time quantitative PCR (RT-qPCR)

Protein extracts from cells and tissues were analyzed for NOS3 (1:500, catalog number 610297, BD Biosciences), p-NOS3 (1:100, catalog number sc-81510, Santa Cruz distributed by CliniSciences, Nanterre - France), and β-actin (1:5,000, catalog number A3854, Sigma-Aldrich). *Plgf* mRNA expression levels were measured by RT-qPCR as previously described.[27]

Statistical analyses

Categorical variables are expressed as numbers (n) and relative frequencies (%), whereas continuous variables are expressed as mean ± SEM. Normality was assessed with the Shapiro-Wilk test. Circulating PlGF levels among healthy controls, patients with cirrhosis and patients with extrahepatic portal hypertension were compared using a one-way ANOVA followed by a Tukey post hoc test. We then evaluated whether circulating PlGF levels were influenced by the severity of cirrhosis by comparing patients based on their Child-Pugh or MELD scores using an unpaired t test. To assess the association between PlGF levels and HPS in patients with cirrhosis, a multivariate logistic regression was performed, after adjustment for liver disease severity assessed by Child-Pugh or MELD scores. Finally, Pearson's correlation coefficient was used to examine the correlation between PlGF levels and the severity of hypoxemia (A-aO_2) in patients with cirrhosis with HPS.

We hypothesized that PlGF levels may be dysregulated in HPS experimental models and that deleting *Plgf* could mitigate HPS severity. In animal models, we compared continuous variables in Sham, CBDL, and PPVL rats (both WT and *Plgf*-deficient rats) using a one-way ANOVA followed by a Tukey post hoc test.

For *in vitro* experiments with human PMECs, we evaluated the effects of two doses of recombinant PlGF stimulation using a one-way repeated measures ANOVA. We assessed the impact of siRNA-mediated inhibition of PlGF using a paired t test.

Statistical analyses were conducted using GraphPad Prism version 10.0.0 for Windows (GraphPad Software), except for the logistic regression, which was performed using IBM SPSS Statistics, version 29 (IBM, Paris, France). Results with p <0.05 were considered statistically significant.

Results

Elevated PlGF levels correlate with hypoxemia in patients with cirrhosis with HPS

Patient demographics, etiologies, and liver disease severity are detailed in Table 1, with healthy subjects serving as controls. Circulating serum PlGF levels were significantly higher in patients with cirrhosis compared with healthy controls (29.4 ± 1.2 *vs.* 20.2 ± 0.8 pg/ml, p <0.0001) (Fig. 1A). Notably, circulating PlGF levels were elevated in patients with cirrhosis, irrespective of the stage of liver disease. There was no significant difference in PlGF levels between patients with Child-Pugh class A cirrhosis and those with Child-Pugh class B or C cirrhosis (30.3 ± 2.3 pg/ml *vs.* 29.0 ± 1.4 pg/ml, respectively) (Fig. 1B). In addition, in patients with Child-Pugh A cirrhosis, those with

Table 1. Demographics and clinical characteristics of control subjects, patients with cirrhosis, and patients with portal cavernoma.

Characteristic	Control	Cirrhosis	Portal cavernoma
Total sample size, n	64	130	7
Age, year, mean ± SEM	44.6 ± 1.6	54.1 ± 1.6	24.9 ± 9.0
Sex, female subjects, n (%)	28 (44)	37 (28)	2 (29)
Child-Pugh			
Class A, n (%)	–	43 (33)	–
Class B or C, n (%)	–	87 (67)	–
MELD score, mean ± SEM	–	15.9 ± 0.6	–
Etiology of cirrhosis, n (%)*			
Advanced liver disease	–	78 (60)	–
Non-alcoholic fatty liver disease	–	30 (23)	–
Viral hepatitis	–	18 (14)	–
Biliary atresia	–	12 (9)	–
Cryptogenic cirrhosis	–	6 (5)	–
Other†	–	11 (8)	–
HPS, n (%)	–	19 (15)	3 (42)
Presence of ascites, n (%)	–	69 (53)	0 (0)
Presence of varices, n (%)	–	88 (67)	5 (71)
History of variceal bleeding, n (%)	–	15 (11)	4 (57)
History of encephalopathy, n (%)	–	32 (25)	0 (0)

*Patients with cirrhosis might have more than one etiology.
†Others include: primary biliary cirrhosis (n = 3), Wilson's disease (n = 2), chronic granulomatous disease (n = 1), type 1 autoimmune hepatitis (n = 1), haemochromatosis (n = 1), ischemic cholangitis (n = 1), sclerosing cholangitis (n = 1) and Klatskin tumor (n = 1). HPS, hepatopulmonary syndrome; MELD, model for end-stage liver disease.

clinically significant portal hypertension with esophageal varices or portosystemic collaterals did not exhibit differences in PlGF levels compared with patients without these clinical features (30.9 ± 3.3 *vs.* 29.6 ± 3.1 pg/ml, respectively). Stratifying patients with cirrhosis by MELD score yielded similar results, because PlGF levels did not differ significantly between those with MELD scores <15 and those with scores ≥15 (27.6 ± 1.6 *vs.* 30.8 ± 1.7 pg/ml, respectively). By contrast, patients with portal cavernoma who exhibited no signs of cirrhosis had PlGF levels comparable with those of healthy controls (18.7 ± 1.4 *vs.* 20.2 ± 0.8 pg/ml respectively) (Fig. 1A).

Among the 130 patients with cirrhosis, 19 were diagnosed with HPS. There was no significant difference in PlGF levels between patients with cirrhosis with HPS and those without pulmonary vascular disorder (Fig. 1C). There was no association between serum PlGF levels and the presence of HPS, as assessed by a logistic regression model adjusted for liver disease severity using either the Child-Pugh score (Odds ratio [OR] 0.960; 95% CI 0.908–1.016,) or the MELD score (OR 0.978; 95% CI 0.930–1.028). However, in patients with HPS, Pearson analysis revealed a significant correlation between elevated circulating PlGF levels and increased A-aO_2 (r = 0.61, *p* = 0.005) (Fig. 1D).

PlGF deficiency mitigates CBDL-induced HPS but not PPVL-induced HPS

To investigate the role of PlGF in the development of HPS, we generated $Plgf^{-/-}$ rats using CRISPR-Cas9 technology by deleting a 663-bp sequence in *Plgf*. These $Plgf^{-/-}$ rats were viable and fertile, and did not exhibit any noticeable abnormalities. We then performed CBDL, PPVL, or Sham surgery on these rats (Fig. 2A).

Circulating PlGF levels and tissue expression were assessed using ELISA for the serum and RT-qPCR analysis for lungs and liver tissues from Sham, CBDL, and PPVL rats (both WT and $Plgf^{-/-}$ rats). Under basal conditions (Sham-operated rats), *Plgf* mRNA levels were higher in lung tissues compared with liver tissues. In WT rats, following CBDL surgery, circulating PlGF levels significantly increased, along with hepatic *Plgf* mRNA levels compared with Sham rats, indicating enhanced hepatic production in cirrhotic rats (Fig. 2B,C). However, no changes in lung *Plgf* mRNA levels were observed after CBDL surgery. Following PPVL surgery, circulating PlGF levels, as well as hepatic and pulmonary *Plgf* mRNA levels, were similar to those in Sham-operated rats (Fig. 2B,C). As expected, in *Plgf*-deficient rats, *Plgf* mRNA was undetectable in both lungs and liver across all surgical groups.

The development of HPS was evaluated in each model. In Sham-operated rats (both WT and $Plgf^{-/-}$ rats), no IPVDs were observed via contrast-enhanced echocardiography or systemic fluorescent microspheres analysis following jugular vein injection. In addition, arterial blood gas analysis showed no evidence of hypoxemia (Fig. 2D–F).

Following CBDL surgery, $Plgf^{-/-}$ rats were partially protected from experimental HPS development, as evidenced by a less significant increase in A-aO_2 and fewer detected IPVDs compared with their WT littermates (Fig. 2D–F). However, after

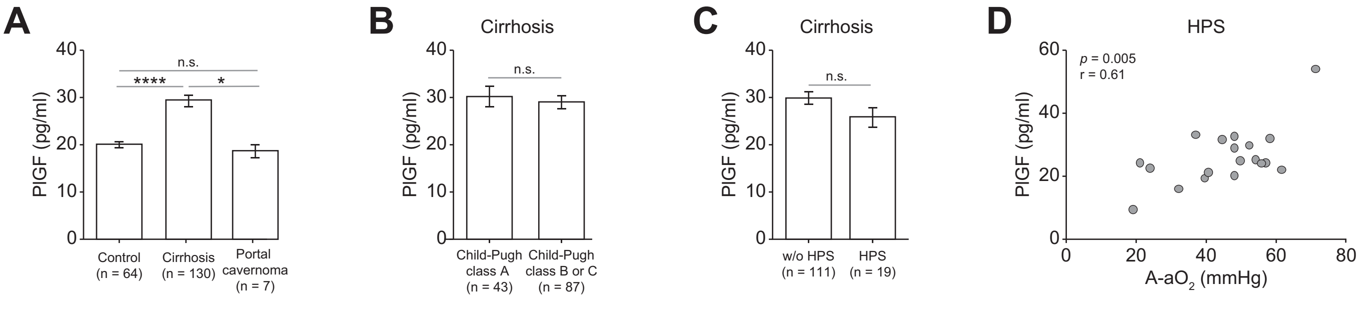

Fig. 1. PlGF levels in humans. (A) Circulating PlGF levels in healthy control subjects, patients with cirrhosis, and patients with portal cavernoma. (B) Among patients with cirrhosis, circulating PlGF levels according to the Child-Pugh class. (C). Among patients with cirrhosis, circulating PlGF levels in patients without and with HPS. (D) Among patients with HPS, correlation between circulating PlGF levels and A-aO_2 assessed by Pearson correlation. Data are mean ± SEM; comparisons were made using one-way ANOVA followed by the Tukey test or by unpaired *t* test. n.s., not significant (*p* >0.05); **p* <0.05, *****p* <0.0001. A-aO_2, alveolar–arterial oxygen gradient; HPS, hepatopulmonary syndrome; PlGF, placental growth factor.

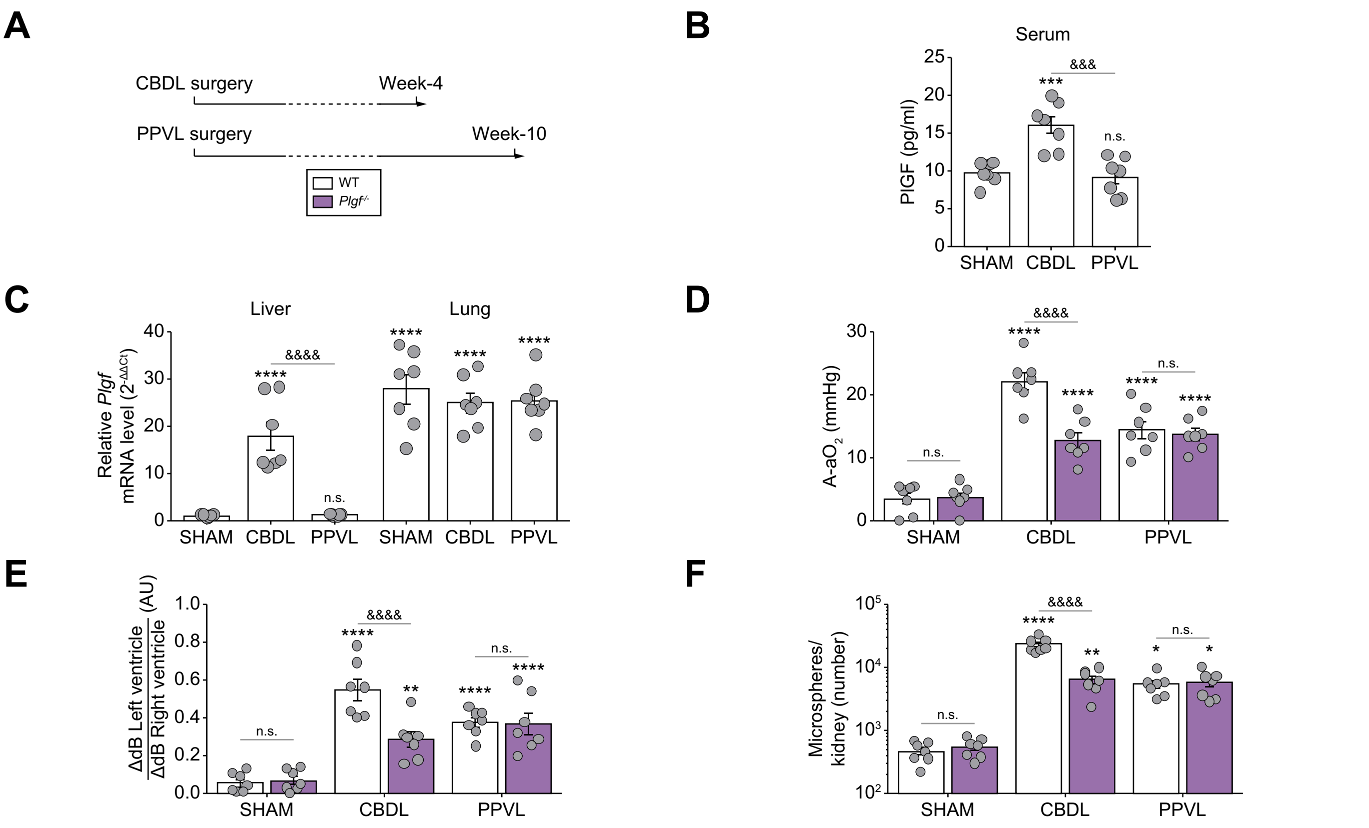

Fig. 2. HPS development in *Plgf*$^{-/-}$ rats after CBDL and PPVL surgery. (A) Experimental procedures. (B) Circulating PlGF levels in serum determined using ELISA. (C) Detection of *Plgf* mRNA levels in liver and lung using RT-qPCR. (D) A-aO_2 in arterial blood. (E) Quantification of contrast-enhanced transthoracic echocardiogram: ratio of the difference in acoustic signal between baseline and after injection in the left ventricle to the right ventricle. (F) Quantification of total microspheres in one kidney by fluorescence-activated cell sorting after jugular vein injection. Data are mean ± SEM; comparisons were made using one-way ANOVA followed by the Tukey test: n.s., not significant (p >0.05); *p <0.05, **p <0.01, ***p <0.001, ****p <0.0001, *vs.* Sham WT group; $^{\&\&\&}p$ <0.001, $^{\&\&\&\&}p$ <0.0001 *vs.* WT CBDL. A-aO_2, alveolar–arterial oxygen gradient; CBDL, common bile duct ligation; HPS, hepatopulmonary syndrome; PlGF, placental growth factor; PPVL, partial portal vein ligation; real-time quantitative PCR (RT-qPCR); WT, wild-type.

PPVL surgery, both *Plgf*$^{-/-}$ rats and their WT littermates developed IPVDs and hypoxemia, with no significant difference in severity between the groups (Fig. 2D–F).

PlGF deficiency attenuates severity of biliary cirrhosis and portal hypertension after CBDL surgery

The development of HPS results from biliary cirrhosis in CBDL rats and from extrahepatic portal hypertension in PPVL rats. We explored surrogate indicators of portal hypertension using hepatic Doppler ultrasound and found that portal venous flow decreased following CBDL surgery. However, *Plgf*$^{-/-}$ rats exhibited a trend towards a less pronounced reduction in portal venous flow compared with WT rats (Fig. 3A). In addition, cirrhotic *Plgf*$^{-/-}$ rats had a less severe increase in liver and spleen weight (Fig. 3B). Following PPVL surgery, the reduction in portal venous flow was similar between *Plgf*$^{-/-}$ rats and their WT littermates, with both groups showing comparable levels of splenomegaly and no hepatomegaly (Fig. 3A,B).

We also investigated liver dysfunction and cirrhosis severity in each model. Interestingly, PlGF deficiency reduced the severity of collagen deposition following cirrhosis induction, as shown by a decrease in the area of Picro-Sirius Red staining in liver sections (Fig. 3C,D). Consistent with these findings, cirrhotic *Plgf*$^{-/-}$ rats had lower levels of aspartate aminotransferase (AST), alanine aminotransferase (ALT), alkaline phosphatase (ALP), and gamma glutamyl transpeptidase (GGT) compared with cirrhotic WT rats (Table 2). Moreover, leukocytosis, commonly observed post CBDL, was less severe in *Plgf*$^{-/-}$ rats, with reduced total white blood cells, neutrophils, lymphocytes, and monocytes compared with their WT littermates (Table 2). Given that cirrhosis and leukocytosis did not develop following PPVL surgery, no differences in these parameters were observed between groups.

PlGF-mediated regulation of pulmonary transcriptome in cirrhosis-associated HPS

Following CBDL surgery, PlGF appears to have a significant role in the development of both cirrhosis and HPS. However, in the PPVL model, PlGF does not appear to have a role in the development of portal hypertension or the severity of HPS. Given the increased hepatic production of PlGF in experimental cirrhosis, we explored the molecular pathways through which this overproduction modulates lung pathophysiology in CBDL rats.

To do so, we performed RNA-sequencing analysis on lung tissue from a new surgical series that included six CBDL and 10

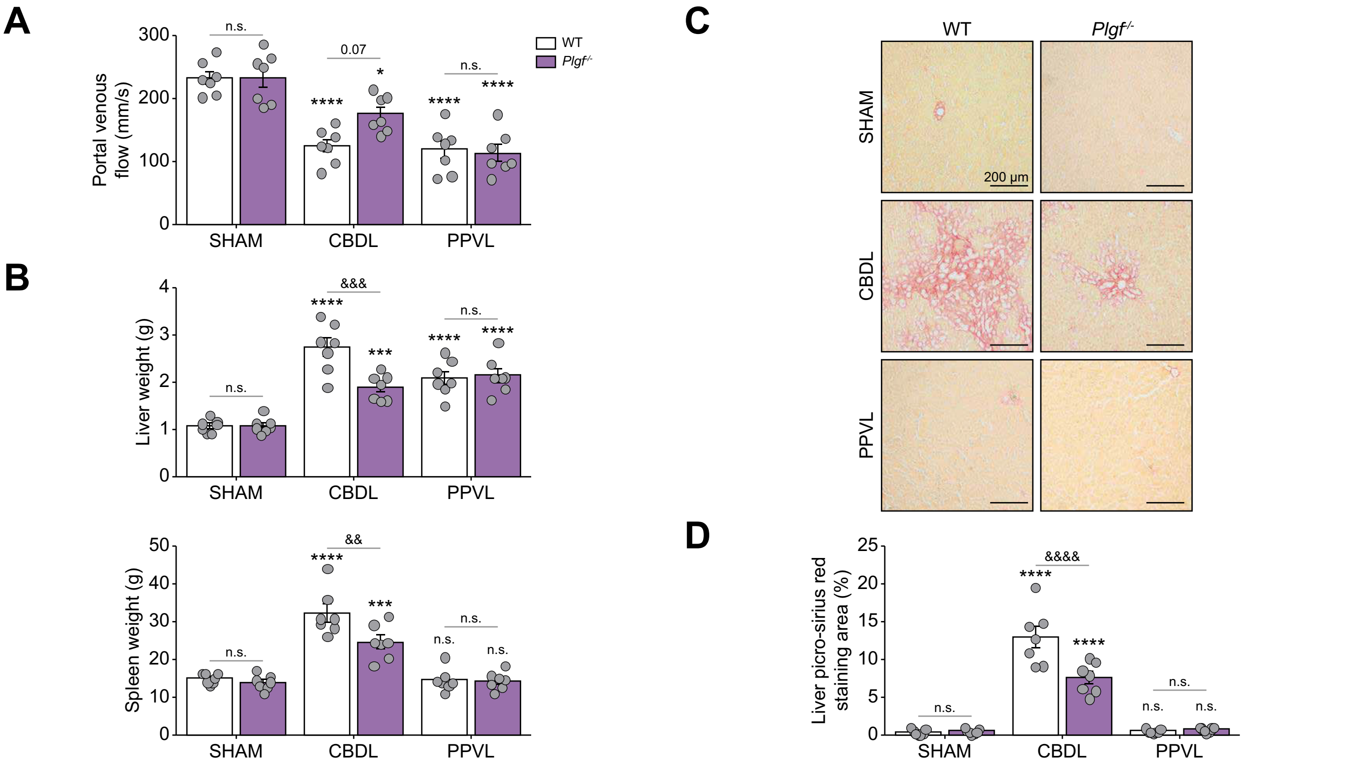

Fig. 3. Portal hypertension and cirrhosis development in *Plgf*$^{-/-}$ rats after CBDL and PPVL surgery. (A) Quantification of portal venous flow by liver Doppler ultrasound. (B) Spleen and liver weight. (C) Picro-Sirius Red staining of liver sections. (D) Quantification of liver fibrotic area by measuring the percentage of Picro-Sirius Red staining. Data are mean ± SEM; Comparisons were made using one-way ANOVA followed by the Tukey test: n.s., not significant (p >0.05); $^{*}p$ <0.05, $^{***}p$ <0.001, $^{****}p$ <0.0001 *vs.* Sham WT group; $^{\&\&}p$ <0.01, $^{\&\&\&}p$ <0.001, $^{\&\&\&\&}p$ <0.0001 *vs.* WT CBDL. CBDL, common bile duct ligation; PlGF, placental growth factor; PPVL, partial portal vein ligation; real-time quantitative PCR (RT-qPCR); WT, wild-type.

SHAM WT rats. This analysis identified 16,626 differentially expressed genes, of which 2,382 were highly significant (p <0.05, fold change >1.5).

We investigated the interactions between PlGF and the pulmonary transcriptome of CBDL rats using Qiagen's IPA. Leveraging the 'My Pathway' tool, we mapped all direct and indirect interactions downstream of PlGF and overlaid our dataset on the generated pathway. PlGF is predicted to modulate several molecules and canonical pathways in the lung (Fig. S2). Notably, our transcriptomic analysis indicated that PlGF was predicted to activate inflammatory and immunomodulatory cytokines, such as CCL4, CXCL8, TNF, CCL28, CCl2, IL6, and IL1B, as well as multiple canonical pathways involved in inflammation.

Interestingly, PlGF is also predicted to activate pathways involved in nitric oxide (NO) production, a key vasodilator with a role in the development of HPS, specifically the pathways identified as 'eNOS signaling' and 'nitric oxide signaling in the cardiovascular system'.

Table 2. Liver biochemistry and complete blood cell count in *Plgf*-deficient rats following surgeries.

	Surgery type					
	Sham		CBDL		PPVL	
**Measurements**	**WT**	***Plgf***$^{-/-}$	**WT**	***Plgf***$^{-/-}$	**WT**	***Plgf***$^{-/-}$
**Liver biochemistry**						
AST (IU/L)	88.1 ± 4.7	77.4 ± 4.2	257.0 ± 19.2‡	181.0 ± 12.3$^{\ddagger,\#}$	84.6 ± 4.7	78.0 ± 7.2
ALT (IU/L)	36.1 ± 1.7	39.1 ± 2.0	80.9 ± 7.6‡	53.7 ± 3.4$^{*,\#}$	48.3 ± 3.3	45.7 ± 3.5
ALP (IU/L)	174.9 ± 9.5	188.6 ± 12.4	352.4 ± 31.9†	261.3 ± 10.1*,§	179.4 ± 17.6	186.3 ± 9.1
GGT (IU/L)	6.4 ± 0.5	6.6 ± 0.7	35.0 ± 5.2†	18.1 ± 2.0$^{*,\#}$	6.6 ± 0.4	6.9 ± 0.5
**Blood cell count**						
White blood cells (10^3/μl)	9.9 ± 0.7	7.0 ± 0.6	34.3 ± 4.2‡	23.4 ± 2.0†,§	9.6 ± 0.6	6.5 ± 0.4
Neutrophils (10^3/μl)	0.9 ± 0.1	0.5 ± 0.1	9.9 ± 1.0‡	6.2 ± 0.6‡,¶	1.0 ± 0.2	0.6 ± 0.1
Lymphocytes (10^3/μl)	8.2 ± 0.4	6.8 ± 0.4	19.5 ± 2.2‡	11.7 ± 0.8$^{\#}$	8.1 ± 0.4	5.7 ± 0.4
Monocytes (10^3/μl)	0.5 ± 0.1	0.3 ± 0.1	6.9 ± 0.7‡	3.2 ± 0.5‡,¶	0.6 ± 0.1	0.2 ± 0.1

Data are mean ± SEM; one-way ANOVA test: $^{*}p$ <0.05, $^{\dagger}p$ <0.001, $^{\ddagger}p$ <0.0001, versus SHAM group; $^{\S}p$ <0.01, $^{\#}p$ <0.001, $^{\P}p$ <0.0001 CBDL *Plgf*$^{-/-}$ *vs.* CBDL WT group. AST, aspartate aminotransferase; ALT, alanine aminotransferase; ALP, alkaline phosphatase; CBDL, common bile duct ligation; GGT, gamma glutamyl transpeptidase; PlGF, placental growth factor; PPVL, partial portal vein ligation; WT, wild-type.

Modulation of pulmonary eNOS activity by PlGF abundance

To investigate the relationship between PlGF and pulmonary eNOS signaling in cirrhotic rats, we measured the levels of eNOS and its phosphorylated form (p-eNOS) at Serine 1177, which is known to activate NO production, in lung homogenates. Following CBDL surgery, we observed increased levels of both p-eNOS and total eNOS in WT rats. Interestingly, in *Plgf*$^{-/-}$ rats, there was a significant decrease in the pulmonary levels of p-eNOS and eNOS, which were normalized compared with Sham WT rats (Fig. 4A). In addition, we examined the levels of NO metabolites (nitrite/nitrate) in the serum of rats following CBDL surgery. Whereas circulating nitrite/nitrate levels were elevated in cirrhotic WT rats, the increase was less significant in *Plgf*$^{-/-}$ rats (Fig. 4B).

To establish a direct link between PlGF and NO production, we performed *in vitro* experiments using primary cultures of human PMECs from six different lung specimens. Cells were stimulated with PlGF at concentrations of 50 ng/ml and 200 ng/ml, and the expression of eNOS and its phosphorylated form on Serine 1177 were analyzed. Interestingly, 30 min of stimulation induced strong phosphorylation of eNOS without altering its total form. However, after 24 h, both p-

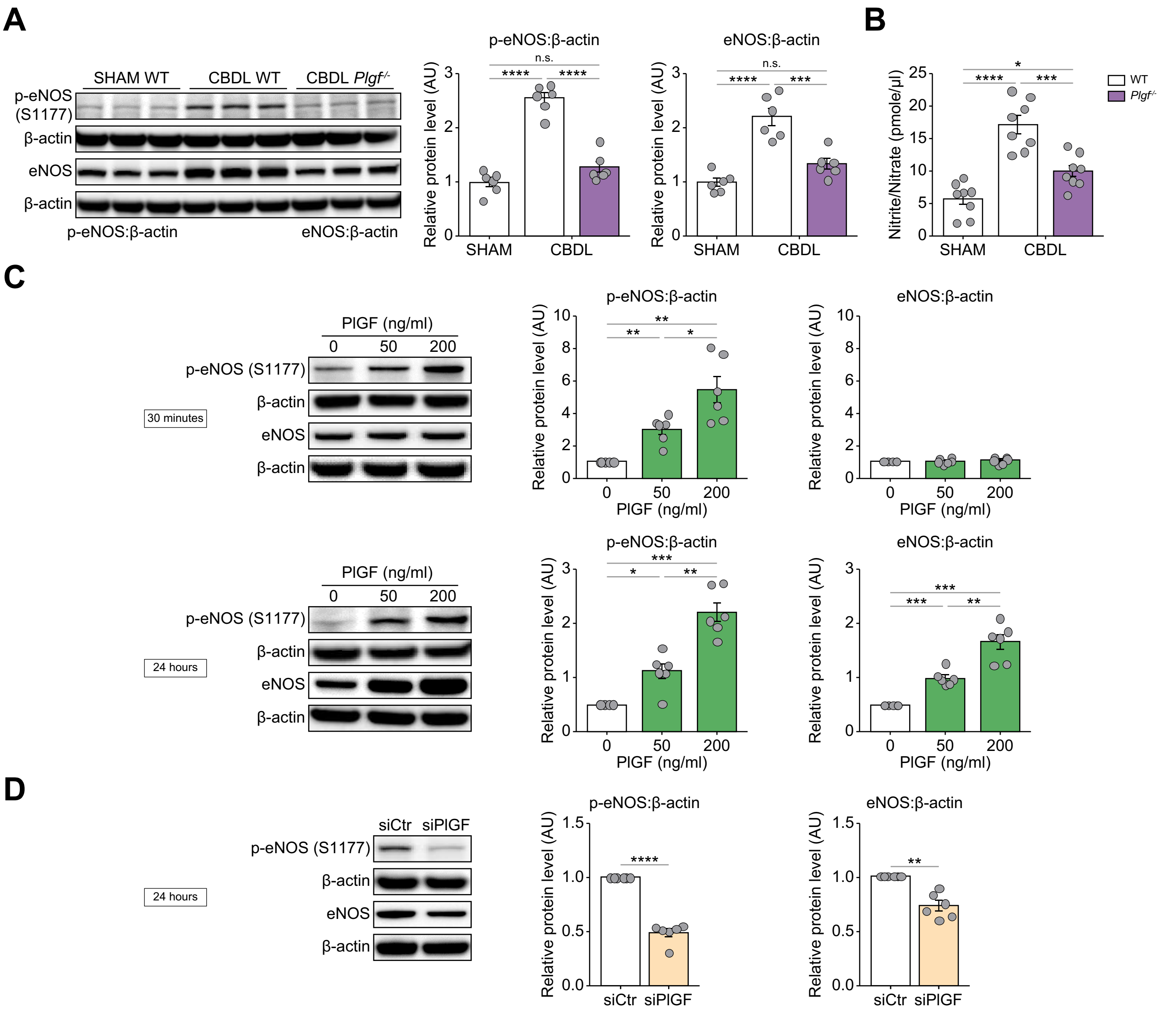

Fig. 4. eNOS activity modulation by PlGF abundance in CBDL rats and in human PMECs. (A) eNOS and p-eNOS expression in WT and *Plgf*$^{-/-}$ rats after CBDL surgery analyzed by western blot. (B) NO metabolites in WT and *Plgf*$^{-/-}$ rats after CBDL surgery. (C) eNOS and p-eNOS expression after 30 min or 24 h of PlGF stimulation (0, 50, or 200 ng/ml). (D) eNOS and p-eNOS expression after transfection with siPlGF or scrambled sequence. Data are mean ± SEM; comparisons were made using one-way ANOVA followed by the Tukey test or by unpaired *t* test: n.s., not significant (p >0.05); *p <0.05, **p <0.01, ***p <0.001, ****p <0.0001. CBDL, common bile duct ligation; eNOS, endothelial nitric oxide synthase; NO, nitric oxide; p-eNOS, phosphorylated eNOS; PlGF, placental growth factor; PMECs, pulmonary microvascular endothelial cells; siPlGF, siRNA-mediated knockdown of PlGF; WT, wild-type.

eNOS and total eNOS levels increased in PMECs (Fig. 4C). To investigate the impact of P1GF inhibition on eNOS activity, we conducted siRNA-mediated knockdown of PlGF (siPlGF) and examined eNOS activity 24 h after transfection. We confirmed the successful suppression by RT-qPCR with the absence of PlGF mRNA detection. The use of siPlGF treatment on PMECs significantly decreased both p-eNOS and eNOS levels (Fig. 4D).

Discussion

Our study provides new insights into the role of PlGF in HPS associated with various types of liver disease, with or without cirrhosis. Consistent with prior reports, we observed significantly elevated PlGF levels in patients with cirrhosis.[33,34] Notably, as previously demonstrated,[35] PlGF levels were comparable between patients with cirrhosis with or without HPS. However, among patients with HPS, higher PlGF levels correlated with more severe hypoxemia. Interestingly, PlGF levels remained unchanged in patients with extrahepatic portal hypertension without cirrhosis, despite the potential for triggering HPS. These observations suggest that, although PlGF exacerbates cirrhosis-induced HPS, it does not directly initiate the condition.

These findings in humans align with those in our rodent studies. Biliary cirrhosis induced by CBDL in rats led to a marked overproduction of PlGF in the liver, resulting in increased circulating PlGF levels. Conversely, in the model of extrahepatic portal hypertension following PPVL surgery, in which we previously demonstrated the development of HPS,[27] circulating PlGF levels, and hepatic and pulmonary PlGF expression remained unaltered.

The role of PlGF in HPS induced by CBDL in mice has been previously highlighted.[22] The administration of anti-PlGF antibodies, both prophylactically and therapeutically, improved the pulmonary phenotype by reducing IPVDs and hypoxemia, attributed to a reduction in both angiogenesis and inflammation. Our findings using *Plgf*-deficient rats post CBDL echoed these results, showing protection against HPS development, albeit without completely reversing the condition. However, the severity of HPS in the long-term PPVL model was comparable between *Plgf*-deficient and WT rats, indicating that PlGF does not contribute to the development of HPS in the context of extrahepatic portal hypertension.

The exacerbation of cirrhosis-induced HPS by PlGF appears to mediated primarily through its modulation of inflammation, angiogenesis,[22] and vascular tone. PlGF is a key circulating factor that regulates the secretion of inflammatory and immunomodulatory cytokines from various cell types.[20–22] Our transcriptomic study revealed the potential role of PlGF in pulmonary inflammation, a crucial component of the CBDL-induced HPS model. We found that PlGF activated inflammatory and immunomodulatory cytokines, such as CCL4, CXCL8, TNF, CCL28, CCL2, IL6, and IL1B, along with canonical pathways involved in inflammation. Moreover, PlGF was predicted to regulate NO production, a pivotal actor in vascular tone modulation, which is strongly implicated in HPS.[36–39]

The effect of PlGF on systemic vascular tone is well documented, particularly in pre-eclampsia, where low circulating PlGF levels predict disease occurrence.[40] PlGF administration has been shown to treat and reverse pre-eclampsia in experimental models. Numerous studies demonstrated a dose-dependent vasodilator effect of PlGF on systemic circulation.[9–14] Interestingly, the systemic vasodilatory effect of PlGF involves NO, given that this effect is reversible with a NO synthase inhibitor.[9,13] However, the role of PlGF in pulmonary NO synthesis and its impact on pulmonary vascular tone remained unexplored until now.

Our research revealed a dose-dependent activation of eNOS by PlGF in cultured human PMECs, with the opposite effect observed following siRNA-mediated knockdown of PlGF. *In vivo*, cirrhotic WT rats post CBDL surgery exhibited elevated pulmonary eNOS and its phosphorylated form, alongside increased circulating NO metabolites, all contributing to HPS development. By contrast, cirrhotic *Plgf*$^{-/-}$ rats displayed normalized levels of pulmonary eNOS, phosphorylated eNOS, and NO metabolites, suggesting a direct role for PlGF in pulmonary NO production.

Finally, we cannot rule out that the beneficial effect of PlGF deficiency on the development of cirrhosis could influence the severity of HPS. Previous studies showed that PlGF inhibition improves cirrhosis severity and liver function across different models.[23–25] Our study similarly found that deleting *Plgf* in rats attenuated CBDL-induced cirrhosis severity, as evidenced by reduced portal hypertension, decreased collagen deposition, and improved liver function in *Plgf*-deficient rats. Finally, future studies are needed to determine the precise role of PlGF on liver cells, including liver sinusoidal endothelial cells.

In conclusion, our study highlights the complex role of PlGF in HPS, particularly in the context of cirrhosis. Whereas elevated circulating PlGF exacerbates cirrhosis-associated HPS severity through its effects on pulmonary vascular tone, inflammation, and angiogenesis, it does not directly trigger the condition. These findings deepen our understanding of the molecular mechanisms underlying HPS and suggest potential therapeutic strategies, particularly through modulation of PlGF activity in patients with cirrhosis. These discoveries offer promising opportunities for improving HPS management by elucidating the interactions between circulating factors and liver pathologies.

Affiliations

[1]*Université Paris-Saclay, Unité Mixte de Recherche en Santé (UMR_S) 999 Hypertension Pulmonaire: Physiopathologie et Innovation Thérapeutique (HPPIT), Le Kremlin-Bicêtre, France;* [2]*INSERM, UMR_S 999 Hypertension Pulmonaire: Physiopathologie et Innovation Thérapeutique (HPPIT), Le Kremlin-Bicêtre, France;* [3]*Department of Translational Medical Sciences, Federico II University, Naples, Italy;* [4]*Université Paris-Saclay, Centre de Ressource Biologique Paris-Saclay, Assistance Publique-Hôpitaux de Paris (APHP), Hôpital Bicêtre, Le Kremlin Bicêtre, France;* [5]*Centre d'Immunophénomique (CIPHE), Aix Marseille Université, INSERM, CNRS, CELPHEDIA, PHENOMIN, Marseille, France;* [6]*Janvier-Labs, France;* [7]*Service de Pneumologie et Soins Intensifs Respiratoires, Centre de Référence de L'hypertension Pulmonaire (PulmoTension), AP-HP, Hôpital Bicêtre, Le Kremlin-Bicêtre, France;* [8]*Centre Hépato-Biliaire, AP-HP, Hôpital Paul Brousse, Villejuif, France;* [9]*INSERM UMR_S 1193, Hepatinov, University Paris-Saclay, Orsay, France;* [10]*Pediatric Hepatology and Liver Transplantation Unit, National Reference Centre for Biliary Atresia and Genetic Cholestasis, AP-HP, Hôpital Bicêtre, Le Kremlin-Bicêtre, France*

Abbreviations

ΔdB, difference in decibels; A-aO_2, alveolar–arterial oxygen gradient; ALP, alkaline phosphatase; ALT, alanine aminotransferase; AST, aspartate aminotransferase; CBDL, common bile duct ligation; eNOS, endothelial nitric oxide synthase; GGT, gamma glutamyl transpeptidase; HPS, hepatopulmonary syndrome; IPA, ingenuity pathway analysis; IPVDs, intrapulmonary vascular dilations; MELD, model for end-stage liver disease; NGS, next-generation sequencing; NO, nitric oxide; OR, odds ratio; p-eNOS. phosphorylated eNOS; $PaCO_2$, partial pressure of arterial carbon dioxide; PaO_2, partial pressure of arterial oxygen; PlGF, placental growth factor; PMECs, pulmonary microvascular endothelial cells; PPVL, partial portal vein ligation; RT-qPCR, real-time quantitative PCR; sgRNA, single guide RNA; siPlGF, siRNA-mediated knockdown of PlGF; siRNA, short interfering RNA; UTR, untranslated region; VEGF, vascular endothelial growth factor; WT, wild-type.

Financial support

This work was supported by funding from the Fondation pour la Recherche Médicale (FRM) grants no. EQU202203014670 (Equipe FRM 2022), the Chancellerie des Universités de Paris (Legs Poix), and the INSERM (Contrat Interface). F.R. is a recipient of a PhD fellowship from the Fondation du Souffle (FdS). A.C. acknowledges funding received from the Heart Failure Association of the ESC in form of an HFA Basic and Translational Research Grant.

Conflicts of interest

Over the past 3 years, C.G. reports grants from Acceleron Pharma, a wholly owned subsidiary of Merck & Co., Inc., MSD, Corteria Pharmaceuticals, Structure therapeutics (ex ShouTi), Diagonal Therapeutics, and Gossamer, outside the submitted work. M.H. reports grants and personal fees from Acceleron, Aerovate, Altavant, AOP Orphan, Bayer, Chiesi, Ferrer, Janssen, Merck, MorphogenIX, and United Therapeutics, outside the submitted work. L.S. reports personal fees from Bayer, MSD, and Janssen, and grants from Acceleron, Janssen, MSD, outside the submitted work. All the other authors declare no conflict of interest regarding the publication of this article.

Please refer to the accompanying ICMJE disclosure forms for further details.

Authors' contributions

Conception and design: FR, LT, CG, LS. Analysis and interpretation: all authors. Drafting manuscript: FR, CG, LS. Generation of *Plgf*$^{-/-}$ rats: FF, F.G.

Acknowledgements

The authors express their gratitude to Axel Perrot for his invaluable assistance with the animals, and to Alexandre Kauskot and Cécile Denis for their expert guidance with flow cytometry. Special thanks are also due to the CIPHE-GEMTIS Consortium investigators, including Fabien Angelis, Corine Arnoux, Thomas Bagarre, Yannis Berkane, Kevin Forin, Cécile Garcia, Robin Lacombe, Naïs Phillippe, and Mireille Richelme, for their essential role in the generation of *Plgf*$^{-/-}$ rats. We also thank the Centre de Ressources Biologiques Paris-Saclay (CRB Paris-Saclay) for the storage of biological samples for pneumology cohort (BRIF Number: BB-0033-00089). We also appreciate the support and collaboration of our laboratory colleagues and the funding agencies that made this research possible.

Supplementary data

Supplementary data to this article can be found online at https://doi.org/10.1016/j.jhepr.2024.101297.

References

[1] Raevens S, Boret M, Fallon MB. Hepatopulmonary syndrome. JHEP Rep 2022;4:100527.
[2] Younis I, Sarwar S, Butt Z, et al. Clinical characteristics, predictors, and survival among patients with hepatopulmonary syndrome. Ann Hepatol 2015;14:354–360.
[3] Schenk P, Schöniger-Hekele M, Fuhrmann V, et al. Prognostic significance of the hepatopulmonary syndrome in patients with cirrhosis. Gastroenterology 2003;125:1042–1052.
[4] Kawut SM, Krowka MJ, Forde KA, et al. Impact of hepatopulmonary syndrome in liver transplantation candidates and the role of angiogenesis. Eur Respir J 2021:2102304.
[5] Fallon MB, Krowka MJ, Brown RS, et al. Impact of hepatopulmonary syndrome on quality of life and survival in liver transplant candidates. Gastroenterology 2008;135:1168–1175.
[6] Baiges A, Turon F, Simón-Talero M, et al. Congenital extrahepatic portosystemic shunts (Abernethy malformation): an international observational study. Hepatology 2020;71:658–669.
[7] Lambert V, Ladarre D, Fortas F, et al. Cardiovascular disorders in patients with congenital portosystemic shunts: 23 years of experience in a tertiary referral centre. Arch Cardiovasc Dis 2021;114:221–231.
[8] Krowka MJ, Fallon MB, Kawut SM, et al. International liver transplant society practice guidelines: diagnosis and management of hepatopulmonary syndrome and portopulmonary hypertension. Transplantation 2016;100:1440–1452.
[9] Osol G, Celia G, Gokina N, et al. Placental growth factor is a potent vasodilator of rat and human resistance arteries. Am J Physiol Heart Circul Physiol 2008;294:H1381–H1387.
[10] Y Tan A, Kearney K, Jenkins C, et al. Cardiovascular and hemodynamic consequences of recombinant placental growth factor administration in Guinea pigs. Hyper Preg 2022;41:99–106.
[11] Makris A, Yeung KR, Lim SM, et al. Placental growth factor reduces blood pressure in a uteroplacental ischemia model of preeclampsia in nonhuman primates. Hypertension 2016;67:1263–1272.
[12] Palei AC, Tan AY, Joo WS, et al. Administration of recombinant human placental growth factor decreases blood pressure in obese hypertensive pregnant rats. J Hypertens 2020;38:2295–2304.
[13] Mandalà M, Gokina N, Barron C, et al. Endothelial-derived hyperpolarization factor (EDHF) contributes to PlGF-induced dilation of mesenteric resistance arteries from pregnant rats. J Vasc Res 2012;49:43–49.
[14] Parenti A, Brogelli L, Filippi S, et al. Effect of hypoxia and endothelial loss on vascular smooth muscle cell responsiveness to VEGF-A: role of flt-1/VEGF-receptor-1. Cardiovasc Res 2002;55:201–212.
[15] Cai J, Ahmad S, Jiang WG, et al. Activation of vascular endothelial growth factor receptor-1 sustains angiogenesis and Bcl-2 expression via the phosphatidylinositol 3-kinase pathway in endothelial cells. Diabetes 2003;52:2959–2968.
[16] Carmeliet P, Moons L, Luttun A, et al. Synergism between vascular endothelial growth factor and placental growth factor contributes to angiogenesis and plasma extravasation in pathological conditions. Nat Med 2001;7:575–583.
[17] Nguyen QD, De Falco S, Behar-Cohen F, *et al.* Placental growth factor and its potential role in diabetic retinopathy and other ocular neovascular diseases. Acta Ophthalmol. 201;96:e1–e9.
[18] Li B, Sharpe EE, Maupin AB, et al. VEGF and PlGF promote adult vasculogenesis by enhancing EPC recruitment and vessel formation at the site of tumor neovascularization. FASEB J 2006;20:1495–1497.
[19] Luttun A, Tjwa M, Moons L, et al. Revascularization of ischemic tissues by PlGF treatment, and inhibition of tumor angiogenesis, arthritis and atherosclerosis by anti-Flt1. Nat Med 2002;8:831–840.
[20] Selvaraj SK, Giri RK, Perelman N, et al. Mechanism of monocyte activation and expression of proinflammatory cytochemokines by placenta growth factor. Blood 2003;102:1515–1524.
[21] Perelman N, Selvaraj SK, Batra S, et al. Placenta growth factor activates monocytes and correlates with sickle cell disease severity. Blood 2003;102:1506–1514.
[22] Raevens S, Geerts A, Paridaens A, et al. Placental growth factor inhibition targets pulmonary angiogenesis and represents a therapy for hepatopulmonary syndrome in mice: liver failure/cirrhosis/portal hypertension. Hepatology 2018;68:634–651.
[23] Li X, Jin Q, Yao Q, et al. Placental growth factor contributes to liver inflammation, angiogenesis, fibrosis in mice by promoting hepatic macrophage recruitment and activation. Front Immunol 2017;8:801.
[24] Li X, Yao Q-Y, Liu H-C, et al. Placental growth factor silencing ameliorates liver fibrosis and angiogenesis and inhibits activation of hepatic stellate cells in a murine model of chronic liver disease. J Cell Mol Med 2017;21:2370–2385.
[25] Van Steenkiste C, Ribera J, Geerts A, et al. Inhibition of placental growth factor activity reduces the severity of fibrosis, inflammation, and portal hypertension in cirrhotic mice. Hepatology 2011;53:1629–1640.
[26] Fallon MB, Abrams GA, McGrath JW, et al. Common bile duct ligation in the rat: a model of intrapulmonary vasodilatation and hepatopulmonary syndrome. Am J Physiol Gastrointest Liver Physiol 1997;272:G779–G784.
[27] Robert F, Certain M-C, Baron A, et al. Disrupted BMP-9 signaling impairs pulmonary vascular integrity in hepatopulmonary syndrome. Am J Respir Crit Care Med 2024;210:648–661.
[28] Abrams GA, Jaffe CC, Hoffer PB, et al. Diagnostic utility of contrast echocardiography and lung perfusion scan in patients with hepatopulmonary syndrome. Gastroenterology 1995;109:1283–1288.

[29] Chojkier M, Groszmann RJ. Measurement of portal–systemic shunting in the rat by using gamma-labeled microspheres. Am J Physiol Gastrointest Liver Physiol 1981;240:G371–G375.
[30] Easter DW, Wade JB, Boyer JL. Structural integrity of hepatocyte tight junctions. J Cell Biol 1983;96:745–749.
[31] Liao Y, Smyth GK, Shi W. The R package Rsubread is easier, faster, cheaper and better for alignment and quantification of RNA sequencing reads. Nucleic Acids Res 2019;47:e47.
[32] Phan C, Jutant E-M, Tu L, et al. Dasatinib increases endothelial permeability leading to pleural effusion. Eur Respir J 2018;51:1701096.
[33] Gelman S, Salteniene V, Pranculis A, et al. Plasma Nogo-A and placental growth factor levels are associated with portal hypertension in patients with liver cirrhosis. World J Gastroenterol 2019;25:2935–2946.
[34] Krawczyk M, Zimmermann S, Hess G, et al. Panel of three novel serum markers predicts liver stiffness and fibrosis stages in patients with chronic liver disease. PLoS ONE 2017;12:e0173506.
[35] Raevens S, Coulon S, Van Steenkiste C, et al. Role of angiogenic factors/cell adhesion markers in serum of cirrhotic patients with hepatopulmonary syndrome. Liver Int 2015;35:1499–1507.
[36] Fallon M, Abrams G, Luo B, et al. The role of endothelial nitric oxide synthase in the pathogenesis of a rat model of hepatopulmonary syndrome. Gastroenterology 1997;113:606–614.
[37] Nunes H, Lebrec D, Mazmanian M, et al. Role of nitric oxide in hepatopulmonary syndrome in cirrhotic rats. Am J Respir Crit Care Med 2001;164:879–885.
[38] Rolla G, Brussino L, Colagrande P, et al. Exhaled nitric oxide and impaired oxygenation in cirrhotic patients before and after liver transplantation. Ann Intern Med 1998;129:375.
[39] Rolla G, Brussino L, Colagrande P, et al. Exhaled nitric oxide and oxygenation abnormalities in hepatic cirrhosis. Hepatology 1997;26:842–847.
[40] Chau K, Hennessy A, Makris A. Placental growth factor and pre-eclampsia. J Hum Hypertens 2017;31:782–786.

Keywords: Liver cirrhosis; Portal hypertension; Pulmonary endothelial dysfunction; Partial portal vein ligation; Common bile duct ligation; Intrapulmonary vascular dilations; Hypoxemia; VEGF.

Received 21 June 2024; received in revised form 27 November 2024; accepted 5 December 2024; Available online 10 December 2024

Journal of Hepatology, Volume *7*

Supplemental information

Placental growth factor modulates endothelial NO production and exacerbates experimental hepatopulmonary syndrome

Fabien Robert, Feriel Benchenouf, My Ngoc Ha, Alessandra Cuomo, Mina Ottaviani, Maxime Surbier, Raphaël Thuillet, Corinne Normand, Florent Dumont, Céline Verstuyft, Frederic Fiore, Frederic Guinut, Marc Humbert, Audrey Coilly, Emmanuel Gonzales, Olivier Sitbon, Ly Tu, Christophe Guignabert, and Laurent Savale

Placental growth factor modulates endothelial NO production and exacerbates experimental hepatopulmonary syndrome

Fabien Robert, Feriel Benchenouf, My Ngoc Ha, Alessandra Cuomo, Mina Ottaviani, Maxime Surbier, Raphaël Thuillet, Corinne Normand, Florent Dumont, Céline Verstuyft, Frederic Fiore, Frederic Guinut, Marc Humbert, Audrey Coilly, Emmanuel Gonzales, Olivier Sitbon, Ly Tu, Christophe Guignabert, Laurent Savale

Table of contents

Fig. S1……………………………………………………………………………………2

Fig. S2……………………………………………………………………………………3

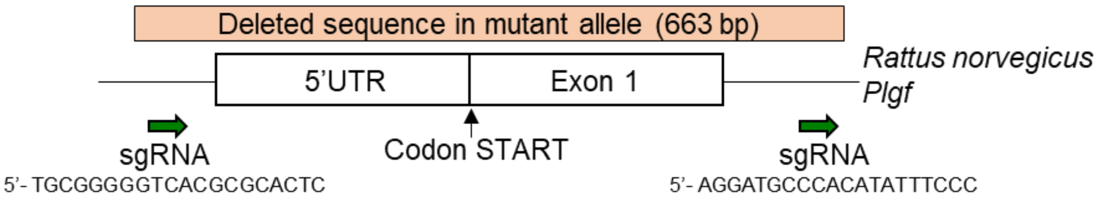

Fig. S1. Generation of the *Plgf*-deficient rat model.

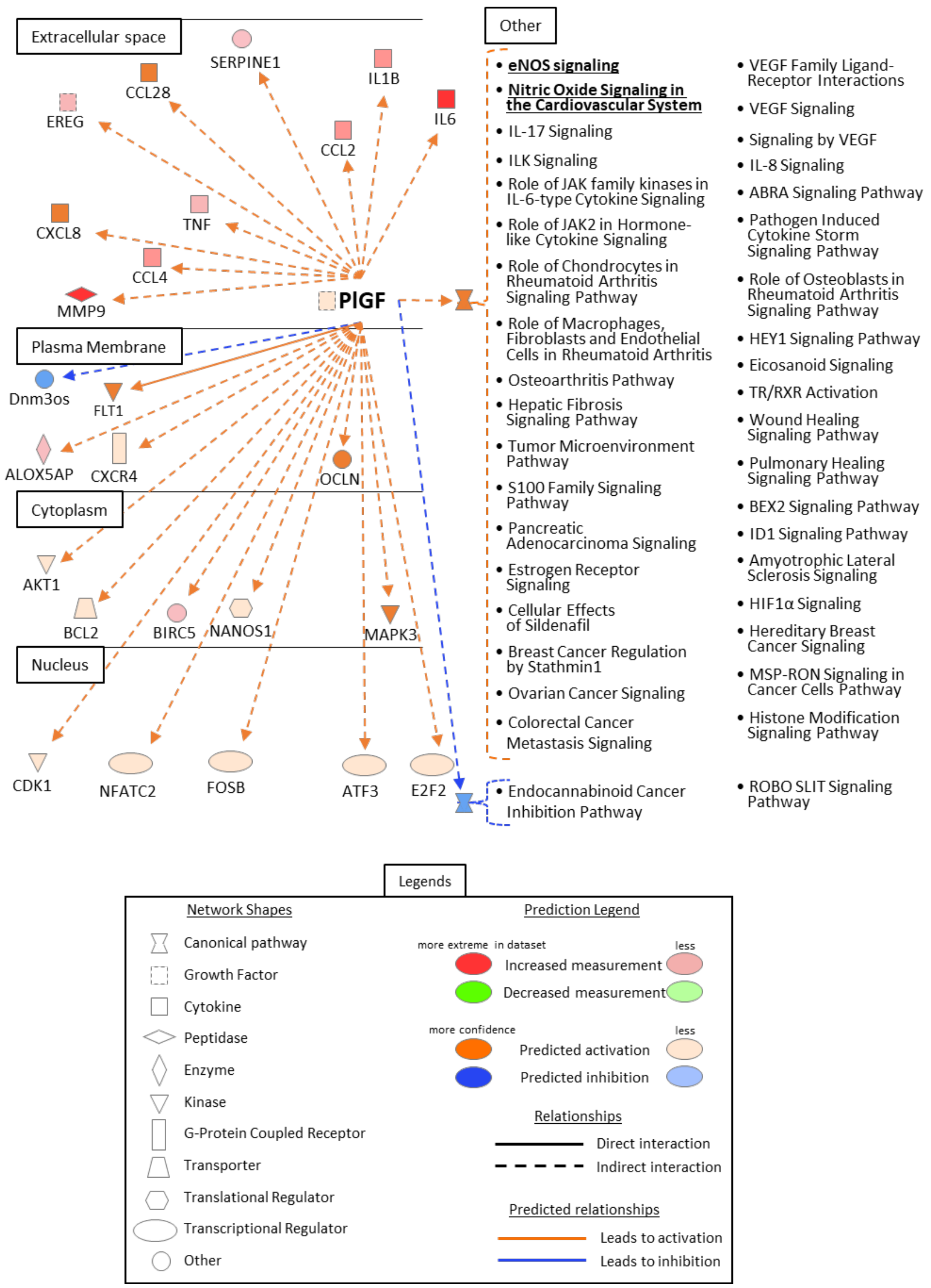

Fig. S2. Interaction between PlGF and the pulmonary transcriptome of CBDL rats using My Pathway analysis (IPA tool, QIAGEN). Cut-off: p-value<0.05, fold-change>|1.5|. The predicted relationships are represented with either full-lined arrows for direct or dotted arrows for indirect interaction. The predicted relationships that lead to activation or inhibition of the downstream targets or gene sets are shown in orange and blue, respectively. Upregulated genes are represented in red and downregulated genes in green. Predicted activated or inhibited genes and gene sets are shown in orange and blue, respectively.
